# Use of Lecanemab for Alzheimer's Disease within the Veteran's Health Foundation: Early Findings

**DOI:** 10.1002/alz70860_101251

**Published:** 2025-12-23

**Authors:** Alison J. O'Donnell, Xinhua Zhao, Alyssa Parr, Sherrie Aspinall, Timothy S. Anderson

**Affiliations:** ^1^ VA Pittsburgh Healthcare System, Pittsburgh, PA, USA; ^2^ University of Pittsburgh, Pittsburgh, PA, USA; ^3^ VA Center for Medication Safety, Washington, DC, USA

## Abstract

**Background:**

Lecanemab, an anti‐amyloid monoclonal antibody for Alzheimer's disease, was approved for use in the Veteran's Health Administration (VHA) in 2023. Little is known about the real‐world use and outcomes of lecanemab.

**Methods:**

This retrospective cohort study included Veterans who initiated lecanemab in the VHA between October 2023 and September 2024. Data on demographics, healthcare utilization, medication exposures, and neuroimaging were extracted from the VA Corporate Data Warehouse. Chart review was used to collect information on dementia diagnosis, Montreal Cognitive Assessments (MoCA), and Apolipoprotein E (APOE) genotype. We examined medication adherence, brain MRIs, 6‐month MoCAs, amyloid‐related imaging abnormalities (ARIA), and healthcare utilization from initiation to study end (12/31/24).

**Results:**

Overall, 32 Veterans (mean [SD] age 75 [6] years, 100% male, 97% white, 84% urban‐dwelling) initiated lecanemab; 53% had mild cognitive impairment and 47% mild dementia. The baseline mean MoCA score was 21 (SD 3). Half were heterozygous for APOE ε4 and half non‐carriers. The median days of follow‐up was 172 (range 62‐349), and the median number of lecanemab infusions received was 11 (2‐24). Brain MRI follow‐up occurred regularly, with all eligible patients receiving an MRI between the 4th and 5th infusion, 92% (22/24) between the 6th and 7th infusions, and 92% (11/12) between the 13th and 14th infusions. However, of the 28 patients with 6 months follow‐up, only 9 (32%) had a MoCA completed and the mean change in MoCA was ‐0.2 (SD 4.2). During follow‐up, 25% of patients had imaging abnormalities: 2 (6.2%) experienced acute stroke and 6 (18.8%) experienced ARIA (3 ARIA‐edema, 3 ARIA‐hemorrhage, and 1 both). Three patients (9.4%) stopped lecanemab for ≥30 days by the end of the study period. Reasons for discontinuation included ARIA, side effects, and patient preference. During follow‐up, 0 patients died, 3 (9.4%) were hospitalized within VHA, and 11 (34.4%) had VHA emergency department or urgent care visits.

**Conclusions:**

In the first year lecanemab was available in VHA, the few patients initiated on treatment were mostly white, male, and urban residents. The finding that 25% of patients experienced ARIA or stroke underscores the importance of monitoring the lecanemab safety and effectiveness long‐term.

